# Yoga to prevent mobility limitations in older adults: feasibility of a randomized controlled trial

**DOI:** 10.1186/s12877-018-0988-8

**Published:** 2018-12-12

**Authors:** Erik J. Groessl, Meghan Maiya, Laura Schmalzl, David Wing, Dilip V. Jeste

**Affiliations:** 1 Department of Family Medicine and Public Health, University of California San Diego, 9500 Gilman Dr. #0994, La Jolla, CA 92093 USA; 2 HSR&D, VA San Diego Healthcare System, San Diego, CA USA; 3 UCSD Stein Institute for Research on Aging, La Jolla, CA USA; 4 College of Science and Integrative Health, Southern California University of Health Sciences, Whittier, CA USA; 5 UCSD Exercise and Physical Activity Resource Center (EPARC), La Jolla, CA USA; 6 Department of Psychiatry, University of California San Diego, La Jolla, CA USA

**Keywords:** Mobility, Yoga, Physical function, Feasibility

## Abstract

**Background:**

The loss of mobility during aging impacts independence and leads to further disability, morbidity, and reduced life expectancy. Our objective was to examine the feasibility and safety of conducting a randomized controlled trial of yoga for older adults at risk for mobility limitations.

**Methods:**

Sedentary older adults (*n* = 46; age 60–89) were recruited and randomized to either yoga or a health education comparison group. Yoga sessions (60-min) occurred 2x weekly, and 90-min health education sessions occurred weekly, for 10 weeks. The primary outcomes were recruitment rate, intervention attendance, and retention at assessments. Adverse event rates and participant satisfaction were also measured. Physical performance measures of gait, balance, and strength and self-report outcome measures were administered at baseline and 10-weeks.

**Results:**

Recruitment lasted 6 months. Retention of participants at the 10-week follow-up was high (89% - performance measures; 98% - self-report questionnaires). Attendance was good with 82% of yoga and 74% of health education participants attending at least 50% of the sessions. No serious adverse events were reported. Patient satisfaction with the interventions was high. The mean effect size for the physical performance measures was 0.35 with some over 0.50. The mean effect size for self-report outcome measures was 0.36.

**Conclusions:**

Results indicate that it is feasible to conduct a larger RCT of yoga for sedentary older adults at risk for mobility problems. The yoga and comparison interventions were safe, well accepted, and well attended. Effect sizes suggest yoga may have important benefits for this population and should be studied further.

**Trial Registration:**

ClinicalTrials # NCT03544879; Retrospectively registered 4 June, 2018.

## Background

Maintaining independence is an important goal for most older adults [1–3]. Physical mobility, defined as the ability to walk safely and independently, [4] is one of the most important factors for maintaining functional independence. [5] Impaired mobility often leads to subsequent broader disability involving activities of daily living (ADLs), [5, 6] reduced quality of life (QOL), [7] and increased mortality. [8, 9] Researchers have identified a subgroup of older adults that are at greater risk for developing mobility-related disability [10, 11]. These older adults are more sedentary, walk more slowly, have decreased strength and balance but typically can still perform daily living activities. Behavioral interventions have been linked to improved mobility in older adults, [3, 12, 13] and mind-body interventions such as yoga may produce broader changes and impact multiple health outcomes simultaneously. Compared with some physical activity interventions, yoga may have more impact on balance, core strength, and upper body strength [14]. Yoga also includes deep breathing, relaxation, and focused attention which can improve sleep and have added mental health benefits [15, 16]. Features of yoga such as transportability, home practice, social interaction, and relaxation aspects may increase its appeal to older adults, providing an additional low-cost activity option.

Research on the benefits of yoga for improving mobility and balance in older adults is growing, but to date, few full-scale randomized controlled trials (RCT) have been conducted and published. A pilot RCT conducted in Australia that focused on improving balance and fall prevention in older adults demonstrated that a 12-week yoga program significantly improved balance and mobility measured by 4-m walk times [17]. A United Kingdom (UK)-based pilot study in which older adults were randomized to yoga or an education booklet found that yoga participants had greater improvements in quality of life and the chair-stand portion of the Short Physical Performance Battery (SPPB) [18]. A 2016 systematic review identified six studies measuring some aspect of mobility or balance and concluded that despite limited study quality, yoga likely improved mobility and balance [14]. Other systematic reviews on the benefits of yoga for improving anxiety/depression, [19] cardiovascular disease, [20] executive function [21], and broader quality of life [22] in older adults all conclude that yoga is safe and likely has benefits, but larger high-quality studies are needed. Thus, although many older adults already practice yoga, a more solid evidence-base is needed to identify specific benefits, target specific populations, and promote wider implementation.

**Our objective was to** conduct a pilot randomized controlled trial of yoga for inactive older adults at risk for further mobility disability. Our primary aim was to study the feasibility of conducting a yoga RCT with this population, including the acceptability and safety of a targeted yoga intervention and comparison intervention.

## Methods

### Design

Inactive older adults (*n* = 46) were randomized to either yoga or a parallel health education comparison group. Both interventions lasted 10 weeks. Study outcomes were assessed by blinded assessors using feasibility and acceptability metrics such as rates of recruitment, attendance, and attrition, along with adverse events and participant satisfaction ratings. Health outcomes were measured with physical performance measures and self-report questionnaires at baseline and at 10-weeks. The target sample size was 45–50 participants to study feasibility, resulting in two cohorts of approximately 22–25 per cohort. This was designed to provide 11–13 persons per yoga or health education group.

### Recruitment

Participants were recruited between July and December 2013 using a variety of methods including flyers that were posted at community centers, libraries, cafes, and public posting boards. Participants of The Successful Aging Evaluation (SAGE) Study were notified about the study via mailings. Eligible University of California San Diego (UCSD) Family Medicine patients received recruitment e-mails through a voluntary feature of MyChart. Recruitment materials and study information were described in the monthly newsletter of the Stein Institute for Research on Aging, and on ResearchMatch, and NIH-funded research participation website..

### Screening and enrollment criteria

Potential participants were pre-screened via telephone for age, inactivity, and “willingness to participate” criteria, and if eligible, scheduled for formal screening. Potential participants provided informed consent before they completed the SPPB [11]. Inclusion criteria were selected to follow standards developed in previous multi-site clinical trials [12, 13] and include: a) age 60–89 years; b) self-reported sedentary lifestyle (Have you exercised in the past 3 months?; this includes walking at a brisk pace or regular walking for exercise purposes. If Yes, approximately how many times? (ineligible if they report more than 3 times in 3 months); c) SPPB summary score > 3 and ≤ 8; d) willingness to attend either yoga or health education for 10 weeks; e) willing to complete two assessments; f) residence in San Diego metropolitan area; g) provided a physician-signed health clearance form. Exclusion criteria: a) practiced yoga >2x in the last year; b) life expectancy < 12 months.

### Baseline assessment and randomization

Eligible participants were scheduled for screening, and informed consent in January 2014. If eligible and consented, they completed a baseline assessment and were randomly assigned to either the yoga or health education interventions by the project coordinator using a computer program (1:1 ratio, blocks of 10 to balance groups). Follow-up assessments were conducted in April and May 2014.

### Yoga intervention

The yoga intervention consisted of 2x weekly 60-min sessions for 10 weeks. Sessions occurred at a community yoga studio with access to parking and public transportation. Group sessions (2 cohorts of 10–12 participants each) were led by a certified, experienced (5 years) instructor who underwent 30 h of additional yoga training focused on working with seniors through Silver Age Yoga (http://www.silverageyoga.org/index2.htm). The yoga intervention was based on Silver Age Yoga programs with adaptations for research by study investigators, and is based on principles of Iyengar yoga with modifications to accommodate all levels of ability.

The yoga sessions began with an instructor-led breathing practice. The instructor then led participants through yoga poses at a slow but gradually increasing pace, with chairs used as props when needed. Meditation and breathing were followed by chair poses (15–20 min), standing poses (10–15 min), floor poses (15 min), and a supine resting pose (Savasana; 10 min). A typical class included 20–25 poses out of 73 possible poses included in the Silver Age Yoga method. An instruction manual and home practice manual were created for the study. Participants were encouraged to practice basic poses at home for about 15 min each day, while emphasizing safety.

### Health education intervention

The health education comparison intervention consisted of weekly, 90-min health information workshops conducted in group format. The intervention was held at a university medical center, with access to public transportation and reimbursed parking. The sessions consisted of a 60-min lecture followed by 30 min of discussion. The lecture titles were: Introduction/ Exploring Communication, The Science of Successful Aging, Acupuncture 101: How it Works & What it is Good for, Quality of Life/Quality of Well Being, Fighting Cancer With Your Fork, Forgiveness via Shakespeare’s: A Winter’s Tale, Better Eyesight in Minutes a Day, Brain Fitness, The Importance of Organic Foods/ Organic Gardening, How Dementia Can Be Modified. Lectures were provided by credentialed experts (physicians/psychologists, etc.) and other clinicians. Instructors were asked to minimize discussions of yoga or meditation. Similar comparison interventions have been used in other large behavioral clinical trials [12, 13].

### Retention and attendance

Attendance at yoga sessions and yoga home practice was emphasized by the instructor during yoga sessions. All participants were contacted by study staff if they missed more than one intervention session without explanation, and were contacted a few weeks before the post-assessment to validate contact information and schedule assessments. Additional reminder e-mails or letters were provided preceding all assessments.

### Measures

Feasibility and acceptability measures including recruitment rates, attendance, attrition, and adverse events were tracked by study staff. Attendance of intervention sessions was tracked by a sign-in sheet verified by the coordinator or instructor each week. Yoga practice outside of instructor-led sessions was tracked using weekly home practice logs. Participants indicated which days, the duration, and estimated physical exertion level (low, medium, or high) for each practice. Assessments at baseline and at the end of the intervention consisted of self-report questionnaires that took 30–40 min to complete, followed by physical performance testing administered by trained assessors lasting 40–50 min. Assessors were blinded to intervention condition and participants were reminded to avoid disclosing their intervention assignment to assessors. Participants received a $30 gift card for completing each assessment.

#### Safety monitoring and adverse events

Intervention safety and adverse events were assessed each week in both intervention groups via an adverse event log. Participants that missed more than one intervention session without explanation were contacted by phone to encourage future attendance and assess adverse events.

#### Mobility functioning measures

**Mobility,** was measured with the Short Physical Performance Battery (SPPB) [11]. The SPPB measures time to walk four meters; time to complete five chair-stands; and balance, with higher scores being associated with decreased disability [10, 11]. The balance assessment consists of 3 separate tests of a participant’s ability to maintain balance for up to 10 s with feet side by side, semi-tandem (one heel next to big toe of other foot) and full tandem (heel to toe). The measure has established psychometric properties [23–25] and has been used in a number of large clinical trials with older adults [12, 13, 26].

**Gait and Balance** were assessed using the Limits of Stability (LOS) test [27], the Sensory Organization Test (SOT), [28], the Step Up and Over (SUO) test, and Rhythmic Weight Shift (RWS) test, and via the NeuroCom Smart EquiTest System (www.natus.com). All tests were administered by trained technicians at UCSD Exercise and Physical Activity Resource Center (EPARC) facilities. The LOS measures components of balance and stability related to reaction time, directional control, and the ability to make corrective movements. Reaction Time is a measure of how long it takes to initiate intentional movement toward the target during the two seconds prior to the cue-to-move, less time demonstrating greater responsiveness. Movement velocity indicates the speed of center of gravity (COG) displacement in degrees per second, with higher values signifying quicker movement through the region of stability. The remaining LOS metrics are represented as percentages with values closer to 100 indicating a greater cone of stability and neuromuscular integration.

The SOT assesses the sensory components of balance by measuring postural sway and predicts fall risk [28]. The sensory scores indicate the ability to maintain stability when isolating the visual, vestibular, and somatosensory systems. The preference ratio score indicates participant’s ability to maintain balance in the presence of inaccurate visual cues, [29] relative to a state where all sensory systems remain unchallenged. Scores are represented as an inverse percentage from 0 to 100, with scores closer to 100 indicating greater stability.

The SUO measures gait quality [30] and predicts fall risk, particularly in navigating curbs or climbing and descending stairs. The Lift-Up Index quantifies the maximum lifting force exerted by the leading leg and is expressed as a percentage of the individual’s weight as measured by the force plate, with scores closer to 100% demonstrating greater force. Movement Time, the time required to complete the entire maneuver, is represented in seconds. The Impact Index is a quantification of the maximum vertical impact force as the lagging leg lands on the surface, expressed as a percentage of body weight. Both the Movement Time and Impact Index indicate greater performance with smaller values.

The RWS measures participant ability to rhythmically move between two targets at different speeds [31]. The RWS measures On-Axis Velocity and Directional Control both left to right and forward and backward. The On-Axis Velocity is the speed of the COG displacement in degrees per second during on-axis movement between the test target(s), with greater velocity indicating faster movement through the region of stability. Directional Control measures the degree to which the test subject moves their COG directly toward the target and compares the amount of on-axis movement relative to off-axis movement. It is expressed as a percentage with values closer to 100 indicating more direct movement.

**Grip Strength** was assessed with a hydraulic grip strength dynamometer [32]. The average of two trials for both the left and right hand were used. Predictive validity of hand grip strength has been shown previously for both disability and mortality [32].

#### Self-report questionnaires

Sociodemographic characteristics and comorbidities were assessed with a short questionnaire. Comorbidity questions focused on common medical conditions (10 physical and 2 mental) and the item counts were summed to form a comorbidity index. **Health-Related Quality of Life** was measured with the SF-36 [33]. The measure provides subscales for 8 different domains of HRQOL and summary scores for physical and mental health. **Depression** was assessed using the 10-item Center for Epidemiologic Studies Short Depression Scale (range 0–30) [34]. **Anxiety** was assessed using the Brief Anxiety Inventory, a 21-item measure (range 0–63) with established reliability [35]/validity [36]. **Sleep quality** was measured using the 21-item Pittsburgh Sleep Quality Index (range 0–21) [37]. **Participant Satisfaction** was rated on a 0–10 scale (0 - no satisfaction, 5 - some or medium, 10 = most positive) using five questions about enjoyment with and benefits of participation, instructor quality, getting to know others, and feeling a common bond with others.

### Statistical analysis

Feasibility outcomes for the two experimental groups such as attendance and attrition were examined using independent sample *t*-tests. However, the study was not adequately powered to evaluate hypotheses about group differences. The main goal was to assess the feasibility and acceptability of yoga for preventing mobility problems in older adults. Demographic factors related to attendance and attrition were explored with Pearson correlations and independent sample *t*-tests. Effect sizes between groups were calculated using Cohen’s *d* for the change in physiological and psychosocial outcomes over time, divided by the standard deviation of the change score. Published effect sizes conventions (small effect - *d* = 0.2, medium effect - *d* = 0.5, large effect - *d* = 0.8) were considered for interpretation [38]. To establish a minimum level of feasibility, safety, and intervention acceptance, the following criteria were identified: Recruitment: Ability to recruit 6 participants per month, or at least 50 participants within 10 months, based on the 12-month planned timeline. Attendance: Mean attendance of 50% of intervention sessions based on data indicating that 9–12 classes produced clinical improvement in a previous study [39] Attrition: Retention of 80% or greater at post-intervention assessment based on published guidelines [40]. Satisfaction: Ratings of 6/10 on three main questions (participation enjoyment, perceived benefit, instructor quality) were deemed minimally acceptable to ensure that on average, participants rated the interventions somewhat favorably. Safety: No more than one serious adverse event attributable to either intervention (used in previous study) [41].

## Results

**Fig. 1:**
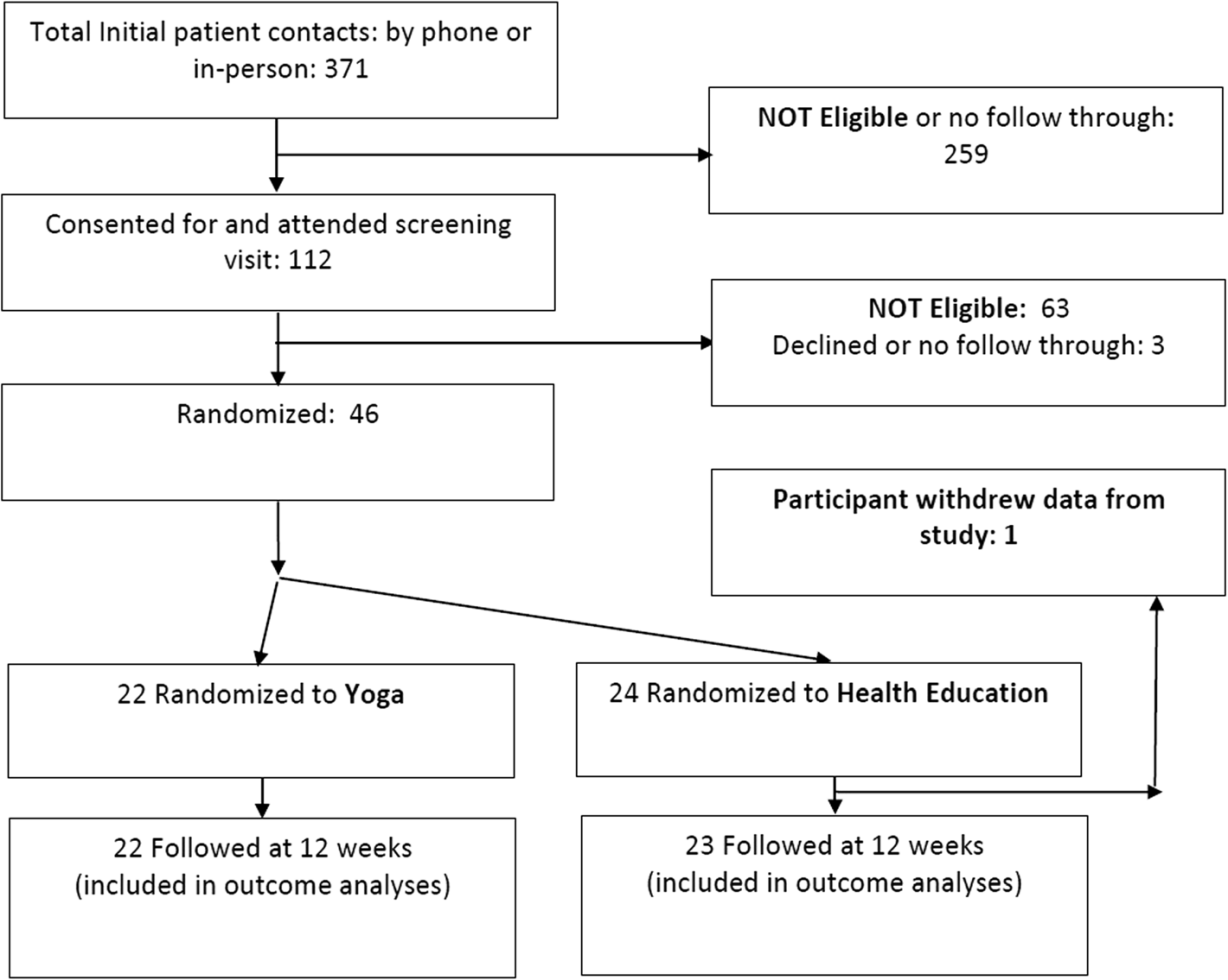
Participant Recruitment and Study Flowchart

A total of 371 people inquired about study participation over the course of six months. (See Fig. 1) After an initial phone pre-screening, 259 were not eligible or declined further screening. The most common reason for ineligibility was recent regular exercise. The remaining 112 individuals were scheduled for a formal screening, at which 63 were found to be ineligible, primarily because they had an SBBP score of 9 or higher. Of the 49 eligible participants, 46 attended a baseline assessment and were randomized to yoga (*n* = 22) or health education (*n* = 24). Self-report questionnaires at the follow-up assessment were completed by 98% (45/46) of participants enrolled, and the physical performance assessment was completed by 89% (41/46) of participants. Participant characteristics are presented in Table 1. When compared to data on all older adults in the recruitment area, non-White minorities were slightly underrepresented in our study (18% vs. 32%) [42]. Gender and education levels were representative of the population.

**Table 1:** Participant Characteristics

		Yoga (*n* = 22); n (%)	Health Education (*n* = 23); n (%)
Demographics			
Age (years)	Mean (SD)	71.6 (8.3)	76.0 (7.8)
Gender (Male or Female)	Female	15 (68%)	13 (57%)
Education (years)	Mean (SD)	15.0	15.8
Race/ethnicity			
African American		0 (0)	1 (4)
White		18 (82)	19 (83)
Native American		1 (4)	0 (0)
Hispanic		3 (14)	2 (9)
	Asian/Pacific Islander	0 (0)	1 (4)
Marital status			
Single		0 (0)	1 (4)
Married		9 (41)	8 (35)
Separated or Divorced		10 (45)	9 (39)
Widowed		3 (14)	5 (22)
Income			
$0-40 K		12 (54)	9 (39)
$40-100 K		8 (36)	13 (56)
$100 K+		1 (5)	0 (0)
Declined		1 (5)	1 (5)
Transportation	Drive Own Vehicle	16 (73)	20 (87)
Ever practiced Yoga? (Yes or No)	No	10 (45)	13 (57)
Health Characteristics	Mean (SD)		
SPPB Total		6.77 (1.38)	6.70 (1.06)
Chair stands		0.73 (.70)	0.74 (0.62)
Gait speed		3.05 (1.05)	3.30 (0.82)
Balance		2.73 (1.35)	2.65 (1.11)
Grip strength (lbs.)		58.2	53.0
Comorbid conditions			
Physical (count 0–10)		2.43 (1.36)	2.43 (1.73)
Mental (count 0–2)		0.57 (0.81)	0.57 (0.73)
SF36			
Physical Functioning		19.3 (5.5)	21.9 (4.1)
Social Functioning		8.5 (3.0)	9.5 (1.8)
General Health		16.5 (3.5)	17.5 (2.2)
Mental Health		22.7 (4.6)	23.9 (4.0)
Physical Role		5.6 (1.6)	6.1 (1.5)
Emotional Role		5.0 (1.3)	5.3 (1.0)
Pain		7.2 (2.5)	7.4 (2.2)
Vitality		13.9 (4.7)	14.7 (3.1)
Mental Component Scale		47.4 (12.7)	50.1 (9.3)
Physical Component Scale		33.9 (8.3)	36.6 (6.2)
Depression (range 0–30)		9.1 (4.2)	7.7 (4.2)
Anxiety (range 0–63)		10.3 (9.2)	7.0 (5.1)
Sleep (range 0–21)		8.2 (4.1)	8.9 (3.9)

*Higher scores indicate better performance or health: SPPB (all scales); Grip Strength; SF36 (all scales)*

*Lower scores indicate better performance or health: co-morbidity index; Depression; Anxiety; Sleep*

*SPPB (0–4 rating for each subscale, total score is sum of 33 subscales 0–12 range)*

*Grip strength (lbs. = pounds of pressure)*

*Co-morbidity index (count of up to 10 physical and 2 mental conditions)*

*SF36 subscales (summed likert scale scores)*

SD = standard deviation

Among the 371 potential participants who expressed interest in the study, the top five recruitment modalities were as follows: UCSD – MyChart (*n* = 175 47%); SAGE Study letter (*n* = 55, 15%); flyers in the community (*n* = 51, 14%); Research Match (*n* = 22, 6%); and SIRA newsletter (*n* = 21, 6%). Recruitment modalities for the 46 participants who enrolled in the study were MyChart (*n* = 13, 28%); flyers (*n* = 12, 26%); SAGE Letter (*n* = 10, 22%); Stein Newsletter (*n* = 5, 11%); Research Match (*n* = 2, 4%); and “word of mouth” or Other (*n* = 4, 9%).The mean number of yoga sessions attended was 14.1 of 20 (71%) sessions which was slightly higher than the mean attendance of 6.0 out of 10 (60%) sessions for health education. When comparing the proportion of participants in each group attending at least half (50%) of the intervention sessions, 82% (18/22) of yoga participants attended at least half of the yoga sessions while 74% (17/23) of the health education participants attended at least half of the health education sessions. Overall attendance was significantly correlated with age (*r* = −.30; *p* = 0.045) suggesting that older age was associated with reduced attendance in both interventions. Attendance was not associated with gender or baseline functioning.

Of the 22 yoga participants, 20 participants (91%) submitted at least one home practice log. The mean number of logs submitted was 8.2 out of 10 (82%). Counting logs not submitted as 0 home practice, participants reported a mean of 3.3 days (sd = 2.0) of home practice totaling a mean of 49 min per week (sd = 42).

**Fig. 2:**
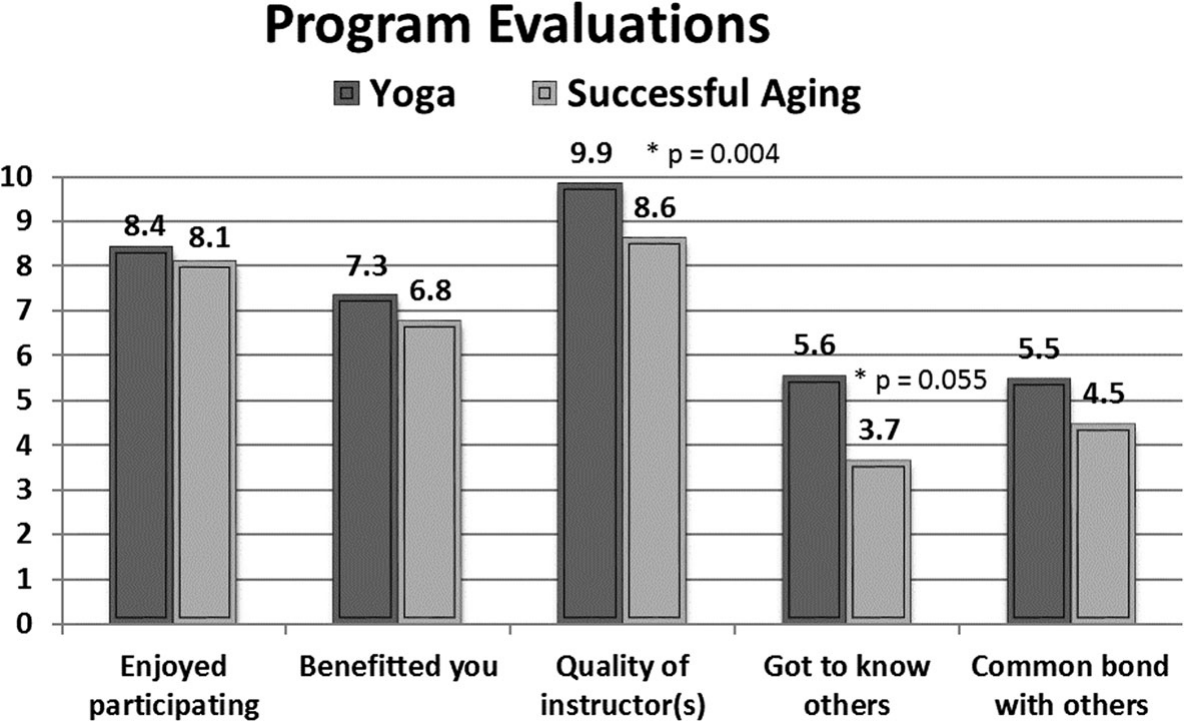
Participant Satisfaction Ratings

No serious adverse events were reported by participants in the study. A total of 22 non-serious adverse events were reported (13 – yoga; 9 – health education). Of the 13 adverse events reported by yoga participants, 6 were considered yoga-related (sore neck [2], arthritis, knee pain, shoulder pain, back pain). In the program satisfaction evaluation, participants rated both interventions very highly, with ratings on a variety of program dimensions presented in Fig. 2. Ratings of instructor quality, satisfaction, and benefits of participation were all high. Social aspects were not a target of the intervention and had moderate ratings on average. Instructor quality and getting to know others was rated significantly higher among yoga participants.

As shown in Table 2, yoga participants tended to have a greater improvement in chair stands on the SPPB than the Health Education participants (Cohen’s *d* = 0.56; 95% C% -0.08 to 1.15). Yoga participants had notably greater improvements than the Health Education participants on five mobility function variables relating to mechanisms of gait and balance (absolute effect size range = 0.38–0.70). The effect size across all physical performance variables was a mean of 0.35, with some exceeding a “moderate” effect size of 0.50. Total grip strength was also better maintained among yoga participants. The mean effect size on self-reported questionnaires of quality of life, mental health, and sleep, was similar (0.36) with improvement favoring the yoga group on all variables (See Table 3). Effects sizes were greater than 0.40 for physical function, social function, emotional-role function, general health, mental health, depression, and anxiety.

**Table 2:** Pre-post change in Physical Performance Measures

Measure	Yoga (*n* = 20)	HC (*n* = 21)	Effect Size- d	95% CI
SPPB Total Score	1.55	1.18	0.26	(− 0.37 to 0.85)
Chair stands	0.85	0.32	0.56	(− 0.08 to 1.15)
Gait	0.40	0.23	0.17	(− 0.43 to 0.78)
Balance	0.60	0.64	− 0.03	(− 0.63 to 0.58)
Grip strength – total (lbs.)	0.09	−9.43	0.63	(− 0.01 to 1.23)
LOS Reaction Time (seconds)	0.14	− 0.01	0.36	(−0.26 to 0.97)
LOS Movement Velocity (degrees/second)	0.51	−0.62	0.70	(0.07 to 1.31)
LOS Endpoint Excursion (%)	9.00	−3.23	0.59	(−0.04 to 1.19)
LOS Maximum Excursion (%)	10.10	−2.50	0.47	(−0.15 to 1.08)
LOS Directional Control (%)	10.90	−11.5	0.61	(−0.03 to 1.21)
SUO Lift (r; %)	−0.70	5.47	−0.18	(−0.77 to 0.43)
SUO Move (r; seconds)	−0.07	8.55	−0.29	(−0.88 to 0.32)
SUO Impact (r; %)	−3.1	6.64	−0.22	(−0.82 to 0.38)
SUO Lift (l; %)	8.71	−0.65	0.48	(−0.13 to 1.08)
SUO Move (l; seconds)	4.75	4.46	0.01	(−0.58 to 0.61)
SUO Impact (l; %)	4.52	5.62	−0.04	(−0.61 to 0.56)
SOT Visual (%)	4.44	4.75	−0.03	(−0.63 to 0.57)
SOT Vestibular (%)	8.90	−1.14	0.38	(−0.23 to 0.98)
SOT Somatosensory (%)	0.75	−1.07	0.36	(−0.25 to 0.95)
SOT Preference (%)	8.58	2.73	0.13	(−0.47 to 0.73)
RWS On Axis Speed (l-r; degrees/second)	−0.15	0.07	−0.22	(−0.81 to 0.39)
RWS Directional (l-r; %)	−4.33	3.77	−0.75	(−1.36 to − 0.12)
RWS On Axis Speed (f-b; degrees/second)	0.02	0.21	−0.31	(−0.90 to 0.30)
RWS Directional (f-b; %)	1.52	6.04	−0.25	(−0.84 to 0.36)

*SPPB* Short Physical Performance Battery, *LOS* Limits of Stability, *SUO* Step Up and Over, *SOT* Sensory Organization Test, *RWS* Rhythmic Weight Shift, *l* left, *r* right, *f* forward, *b* backward

*Measures in which larger change scores indicate improvement: SPPB, Grip Strength, LOS-Movement Velocity, LOS-Endpoint Excursion, LOS-Maximum Excursion, LOS-Directional Control, SUO-Lift, SOT-all scales, RWS-all scales*

*Measures in which smaller change scores indicate improvement: LOS-Reaction Time, SUO-Move, SUO-Impact SPPB (0–4 rating for each subscale, total score is sum of 33 subscales 0–12 range)*

*Grip strength (lbs. = pounds of pressure)*

**Table 3:** Pre-post change in Quality of Life and Functional Outcomes

Measure	Yoga (*n* = 22)	HC (*n* = 23)	Effect - d	95% CI
SF-36 mean change				
Physical Functioning	5.99	−3.70	0.53	(−0.07 to 1.12)
Social Functioning	6.21	−3.73	0.45	(−0.15 to 1.03)
General Health	0.93	−3.14	0.54	(−0.07 to 1.12)
Mental Health	−0.23	−8.04	0.46	(−0.14 to 1.04)
Physical Role	−2.56	−2.95	0.05	(−0.54 to 0.63)
Emotional Role	2.56	−1.72	0.48	(−0.12 to 1.06)
Pain	−2.66	−6.04	0.13	(−0.46 to 0.71)
Vitality	−1.42	−8.15	0.29	(−0.31 to 0.87)
Mental Component Scale	0.66	−3.17	0.57	(−0.04 to 1.16)
Physical Component Scale	0.23	−1.20	0.25	(−0.34 to 0.84)
Depression	−0.35	1.14	−0.44	(−1.04 to 0.18)
Anxiety	0.18	2.87	−0.40	(−1.01 to 0.22)
Sleep	−0.50	−0.20	− 0.13	(− 0.73 to 0.48)

## Discussion

The results of our pilot randomized controlled trial indicate that it is feasible to conduct an RCT of yoga and health education among sedentary older adults at risk for mobility problems using the protocol described above. Despite limited funding, we generally met our recruitment goals and retained 90% of participants at the follow-up assessment. The interventions were well-attended although participants appeared to slightly favor yoga over health education on some intervention dimensions. The yoga and comparison interventions appear safe, with only minimal adverse events being reported.

Study recruitment was more challenging than expected. Although the recruitment methods employed generated 371 potential participants in approximately 6 months, only 49 (13%) met eligibility criteria and 46 enrolled in the study. The main finding was that despite study recruitment materials stating up-front that sedentary older adults were sought, many active older adults inquired because they were interested. Less often, older adults who were sedentary but lived 30–50 miles away inquired in hopes that interventions could be delivered at more distant locations. These factors appear likely to occur in a larger subsequent study, but can be addressed with adequate study funding.

Health outcome data suggest that the yoga group showed more improvement or better maintenance of function on most indicators of mobility including time to stand up from a chair, grip strength, and most performance measures related to gait and balance. These measures are strong indicators of fall risk, [28, 43–45] and are important for maintaining mobility and independence. Self-report measures including health-related quality of life (SF-36) and some mental health measures showed milder but similar effect sizes favoring yoga participants. However, given the limited sample size, the study was not powered to make conclusions about the superiority of yoga for improving health outcomes, and most of the effect size confidence intervals included 0. A larger fully powered trial is being planned to address this question.

A recently published pilot study that randomized older adults to yoga or an informational control group found similar results [18]. Our results confirm their finding that yoga participants had greater improvements on the chair-stand portion of the SPPB. They also found greater improvements in quality of life among yoga participants. However, it is important to note that their control group only received an educational booklet. Methodological research suggests that using an inactive control intervention such as an educational booklet tends to enhance the chances of finding significant differences over time between groups [46] but some of this effect may result from non-intervention-specific factors. The “active control” intervention in our current study involved ten 90-min intervention sessions including lecture and discussion in a supportive environment with peers. Another important way in which our current study can be differentiated from this prior study was the extensive battery of additional physical performance testing. The use of a stronger control intervention and in-depth physical performance testing was chosen to provide a rigorous test of feasibility for conducting a high-quality, impactful full-scale RCT.

Despite high rates of participant retention comparable to those in a recent high-quality pilot RCT of yoga, [47] fewer participants completed the physical performance battery than the self-reported questionnaires (41 versus 45). This suggests that the burden of the performance measures could possibly be reduced or extra care given to support older participants with this assessment. Thus, planning efforts for a full-scale trial will move to recently developed and acquired gait and balance tests with lower burden.

The adverse events experienced were minor. Generalized soreness, including muscle aches and/or mild joint pain in the yoga group was expected, and coincides with findings from other large physical activity trials conducted with older participants [12, 13]. Thus, our preliminary data suggest that instructed yoga sessions and recommended home practice does not appear to pose any more risk than walking in older adults, but these findings should be confirmed in a larger trial.

Patient satisfaction was high in both groups which suggests that the yoga was acceptable and likely to be maintained. Many participants inquired about how and where they could attend other yoga classes after the intervention ended. Some were seeking a low-cost option or free classes while others simply wanted referral to a similar class. The health education sessions were also well attended. Satisfaction ratings slightly favored the yoga group in terms of rating the quality of the instructor and getting to know other people. Instructor ratings were very high in both interventions, with the yoga instructor being rated 9.9. Ratings this high are not necessarily needed nor expected, a future full-scale study should plan to use multiple instructors, and plan for some variability in fidelity, and satisfaction ratings. It is also interesting that participants doing yoga reported that they got to know each other better despite possibly equal or even greater chances to talk and interact during discussion sessions after health education presentations.

Finally, although few conclusions can be made about the differences on health outcomes between groups and the effect sizes found, it was surprising that health education participants reported a sizable improvement on the SPPB after just 10 weeks, and yet reported worse outcomes on many SF36 variables including physical function. It is possible that some lecturers may have provided health behavior motivation in addition to health information during lectures. This suggests that investigators should attend to content in the comparison intervention and should carefully balance delivering an interesting intervention that is well received but does directly change health behaviors that will affect outcomes. It is also possible that some of the increase on outcomes may be attributable to participants remembering test procedures and/or feeling more comfortable during testing.

In addition, some health improvements in the yoga group seemed milder than expected, and many of the 95% confidence intervals were wide and included 0. This may have been due to the emphasis on safety, with all participants practicing yoga while seated in a chair in the early sessions. Not surprisingly, SPPB chair stands was an area where yoga participants clearly improved. Thus, when combined with participant comments in the program evaluation, we believe a longer intervention period is warranted. Participants were disappointed the intervention ended “so quickly”, and an intervention closer to 26 weeks (6 months) long would allow for better elucidation of potential changes in key health metrics in sedentary older participants, particularly those related to mobility, gait, and balance. It is possible that longer interventions are necessary to produce significant change in older participants, with other large studies following this approach [13, 48, 49].

One measure that produced unexpected results was the RWS directional control variable, which indicated larger increases on-axis stability among health education participants. However, the importance of this is unclear. It is possible that yoga participants were less concerned about falling, and thus made less effort to avoid off-axis movement.

The study has a number of limitations. As a feasibility trial with limited funding, the sample size was designed to adequately assess feasibility and not the efficacy or effectiveness of the intervention. The study was conducted at a single site in Southern California, and the two yoga intervention cohorts were led by a single yoga instructor. It is possible that the feasibility and acceptance results may not generalize to other geographical locations, or to other yoga instructors. The frequency of intervention sessions differed between the groups, with yoga sessions being offered twice weekly and the long health education group meeting once per week. Thus, simply getting to the intervention more often could mean more activity for the yoga group. The degree to which study participants did each yoga pose and complied with guidance from the yoga instructor was not measured, limiting objective statements about the intensity of yoga actually practiced. In addition, the study was retrospectively registered with Clinicaltrials.gov.

## Conclusion

A yoga intervention designed to prevent mobility limitations among sedentary older adults was safe and well received by participants. Recruitment, retention, and intervention adherence rates from the pilot RCT indicate that it is feasible to conduct a larger full-scale RCT to study the health benefits of this yoga intervention. The effect sizes found for various physical performance and health outcomes warrant further study. There are also indications that a longer intervention may be beneficial.

## Abbreviations

ADLs: Activities of daily living
COG: Center of gravity
ePARC: Exercise and physical activity tesource center
LOS: Limits of stability
QOL: Quality of life
RCT: Randomized controlled trial
RWS: Rhythmic weight shift
SAGE: Successful aging evaluation
SOT: Sensory organization test
SPPB: Short physical performance battery
SUO: Step up and over
UCSD: University of California San Diego
UK: United Kingdom

## References

[1] Fielding RA, Rejeski WJ, Blair S, Church T, Espeland MA, Gill TM, et al. The lifestyle interventions and Independence for elders study: design and methods. J Gerontol A Biol Sci Med Sci. 2011. 10.1093/gerona/glr123 PMC3193523 21825283

[2] Lonergan ET, Krevans JR (1991). A national agenda for research on aging. N Engl J Med.

[3] Ross LA, Schmidt EL, Ball K. Interventions to maintain mobility: What works? Accid Anal Prev. 2013;61:167–96. 10.1016/j.aap.2012.09.027 PMC3633644 23083492

[4] Patla AE, Shumway-Cook A (1999). Dimensions of mobility: defining the complexity and difficulty associated with community mobility. J Aging Phys Act.

[5] Guralnik JM, LaCroix AZ, Abbott RD, Berkman LF, Satterfield S, Evans DA (1993). Maintaining mobility in late life. I. Demographic characteristics and chronic conditions. Am J Epidemiol.

[6] Fried LP, Bandeen-Roche K, Chaves PH, Johnson BA (2000). Preclinical mobility disability predicts incident mobility disability in older women. J Gerontol A Biol Sci Med Sci.

[7] Groessl EJ, Kaplan RM, Rejeski WJ, Katula JA, King AC, Frierson G (2007). Health-related quality of life in older adults at risk for disability. Am J Prev Med.

[8] Khokhar SR, Stern Y, Bell K, Anderson K, Noe E, Mayeux R (2001). Persistent mobility deficit in the absence of deficits in activities of daily living: a risk factor for mortality. J Am Geriatr Soc.

[9] Newman AB, Simonsick EM, Naydeck BL, Boudreau RM, Kritchevsky SB, Nevitt MC (2006). Association of long-distance corridor walk performance with mortality, cardiovascular disease, mobility limitation, and disability. JAMA.

[10] Guralnik JM, Leveille S, Volpato S, Marx MS, Cohen-Mansfield J (2003). Targeting high-risk older adults into exercise programs for disability prevention. J Aging Phys Act.

[11] Guralnik JM, Simonsick EM, Ferrucci L, Glynn RJ, Berkman LF, Blazer DG (1994). A short physical performance battery assessing lower extremity function: association with self-reported disability and prediction of mortality and nursing home admission. J Gerontol.

[12] Pahor M, Blair SN, Espeland M, Fielding R, Gill TM, Guralnik JM (2006). Effects of a physical activity intervention on measures of physical performance: results of the lifestyle interventions and independence for elders pilot (LIFE-P) study. J Gerontol A Biol Sci Med Sci.

[13] Pahor M, Guralnik JM, Ambrosius WT, Blair S, Bonds DE, Church TS (2014). Effect of structured physical activity on prevention of major mobility disability in older adults: the LIFE study randomized clinical trial. JAMA.

[14] Youkhana S, Dean CM, Wolff M, Sherrington C, Tiedemann A (2016). Yoga-based exercise improves balance and mobility in people aged 60 and over: a systematic review and meta-analysis. Age Ageing.

[15] Groessl EJ, Chopra D, Mills PJ (2015). An overview of yoga research for health and well-being. Journal of Yoga and Physical Therapy.

[16] Halpern J, Cohen M, Kennedy G, Reece J, Cahan C, Baharav A (2014). Yoga for improving sleep quality and quality of life for older adults. Altern Ther Health Med.

[17] Tiedemann A, O'Rourke S, Sesto R, Sherrington C (2013). A 12-week Iyengar yoga program improved balance and mobility in older community-dwelling people: a pilot randomized controlled trial. J Gerontol A Biol Sci Med Sci.

[18] Tew GA, Howsam J, Hardy M, Bissell L (2017). Adapted yoga to improve physical function and health-related quality of life in physically-inactive older adults: a randomised controlled pilot trial. BMC Geriatr.

[19] Klainin-Yobas P, Oo WN, Suzanne Yew PY, Lau Y (2015). Effects of relaxation interventions on depression and anxiety among older adults: a systematic review. Aging Ment Health.

[20] Barrows JL, Fleury J (2016). Systematic review of yoga interventions to promote cardiovascular health in older adults. West J Nurs Res.

[21] Luu K, Hall PA. Hatha yoga and executive function: a systematic review. J Altern Complement Med. 2016;22(2):125–33. 10.1089/acm.2014.0091 26398441

[22] Tulloch A, Bombell H, Dean C, Tiedemann A. Yoga-based exercise improves health-related quality of life and mental well-being in older people: a systematic review of randomised controlled trials. Age Ageing. 2018. 10.1093/ageing/afy044 29584813

[23] Guralnik JM, Ferrucci L, Pieper CF, Leveille SG, Markides KS, Ostir GV (2000). Lower extremity function and subsequent disability: consistency across studies, predictive models, and value of gait speed alone compared with the short physical performance battery. J Gerontol A Biol Sci Med Sci.

[24] Nguyen US, Kiel DP, Li W, Galica AM, Kang HG, Casey VA (2012). Correlations of clinical and laboratory measures of balance in older men and women. Arthritis Care Res (Hoboken).

[25] Penninx BW, Ferrucci L, Leveille SG, Rantanen T, Pahor M, Guralnik JM (2000). Lower extremity performance in nondisabled older persons as a predictor of subsequent hospitalization. J Gerontol A Biol Sci Med Sci.

[26] Bean JF, Herman S, Kiely DK, Frey IC, Leveille SG, Fielding RA (2004). Increased velocity exercise specific to task (InVEST) training: a pilot study exploring effects on leg power, balance, and mobility in community-dwelling older women. J Am Geriatr Soc.

[27] Gyllensten AL, Hui-Chan CW, Tsang WW (2010). Stability limits, single-leg jump, and body awareness in older tai chi practitioners. Arch Phys Med Rehabil.

[28] Panzer VP, Wakefield DB, Hall CB, Wolfson LI (2011). Mobility assessment: sensitivity and specificity of measurement sets in older adults. Arch Phys Med Rehabil.

[29] Pletcher ER, Williams VJ, Abt JP, Morgan PM, Parr JJ, Wohleber MF (2017). Normative data for the NeuroCom sensory organization test in US military special operations forces. J Athl Train.

[30] Boudreau SN, Dwyer MK, Mattacola CG, Lattermann C, Uhl TL, McKeon JM (2009). Hip-muscle activation during the lunge, single-leg squat, and step-up-and-over exercises. J Sport Rehabil.

[31] Delbaere K, Crombez G, Van Den Noortgate N, Willems T, Cambier D (2006). The risk of being fearful or fearless of falls in older people: an empirical validation. Disabil Rehabil.

[32] Rantanen T, Guralnik JM, Foley D, Masaki K, Leveille S, Curb JD (1999). Midlife hand grip strength as a predictor of old age disability. JAMA.

[33] Stewart AL, Ware JE. Measuring Functioning and well-being: the medical outcomes study approach. Durham: Duke University Press; 1992.

[34] Radloff LS (1977). The CES-D scale: a self-report depression scale for research in the general population. Appl Psycho Measures.

[35] Beck AT, Epstein N, Brown G, Steer RA (1988). An inventory for measuring clinical anxiety: psychometric properties. J Consult Clin Psychol.

[36] Fydrich T, Dowdall D, Chambless DL (1992). Reliability and validity of the Beck anxiety inventory. J Anxiety Disord.

[37] Buysse DJ, Reynolds CF, Monk TH, Hoch CC, Yeager AL, Kupfer DJ (1991). Quantification of subjective sleep quality in healthy elderly men and women using the Pittsburgh sleep quality index (PSQI). Sleep.

[38] Cohen J (1988). Statistical power analysis for the behavioral sciences.

[39] Saper RB, Boah AR, Keosaian J, Cerrada C, Weinberg J, Sherman KJ (2013). Comparing once- versus twice-weekly yoga classes for chronic low Back pain in predominantly low income minorities: a randomized dosing trial. Evid Based Complement Alternat Med.

[40] Cramer H, Langhorst J, Dobos G, Lauche R (2015). Associated factors and consequences of risk of Bias in randomized controlled trials of yoga: a systematic review. PLoS One.

[41] Groessl EJ, Liu L, Chang DG, Wetherell JL, Bormann JE, Atkinson JH (2017). Yoga for military veterans with chronic low Back pain: a randomized clinical trial. Am J Prev Med.

[42] County of San Diego, Health & Human Services Agency. Public Health Services. Community health statistics unit. San Diego County senior health report; 2015. p. 10–11.

[43] Brodie MA, Coppens MJ, Ejupi A, Gschwind YJ, Annegarn J, et al. Comparison between clinical gait and daily-life gait assessments of fall risk in older people. Geriatr Gerontol Int. 2017;17(11):2274–82. 10.1111/ggi.12979 28176431

[44] Taylor ME, Ketels MM, Delbaere K, Lord SR, Mikolaizak AS, Close JC (2012). Gait impairment and falls in cognitively impaired older adults: an explanatory model of sensorimotor and neuropsychological mediators. Age Ageing.

[45] Vellas BJ, Wayne SJ, Romero L, Baumgartner RN, Rubenstein LZ, Garry PJ (1997). One-leg balance is an important predictor of injurious falls in older persons. J Am Geriatr Soc.

[46] Park CL, Groessl E, Maiya M, Sarkin A, Eisen SV, Riley K (2014). Comparison groups in yoga research: a systematic review and critical evaluation of the literature. Complementary therapies in medicine.

[47] Ward L, Stebbings S, Athens J, Cherkin D, David Baxter G. Yoga for the management of pain and sleep in rheumatoid arthritis: a pilot randomized controlled trial. Musculoskeletal care. 2018;16(1):39–47. 10.1002/msc.1201 28621011

[48] Cronan TA, Hay M, Groessl E, Bigatti S, Gallagher R, Tomita M (1998). The effects of social support and education on health care costs after three years. Arthritis Care Res.

[49] Wang MY, Greendale GA, Yu SS, Salem GJ (2016). Physical-Performance Outcomes and biomechanical correlates from the 32-week yoga empowers seniors study. Evid Based Complement Alternat Med.

